# 肺多发磨玻璃结节的诊治策略

**DOI:** 10.3779/j.issn.1009-3419.2020.102.10

**Published:** 2020-08-20

**Authors:** 宝东 刘

**Affiliations:** 100053 北京, 首都医科大学宣武医院胸外科 Department of Thoracic Surgery, Xuanwu Hospital, Capital Medical University, Beijing 100053, China

**Keywords:** 肺结节, 磨玻璃结节, 多灶, 诊断, 治疗, Pulmonary nodules, Ground-glass nodule, Multifocal, Diagnosis, Treatment

## Abstract

近年来, 随着高分辨率计算机断层扫描(high resolution computed tomography, HRCT)筛查肺癌项目的开展, 肺多发磨玻璃结节(ground-glass nodule, GGN)发现的越来越多。由于肺多发GGN的诊治目前还存在着许多不确定性, 因此本文对其随访间隔与时限、主病灶和次要病灶的关系、诊断、治疗和残留结节随访等临床关注的相关问题加以综述。

## Abstract (en)

In recent years, with the development of the high resolution computed tomography (HRCT) screening program for lung cancer, the multifocal ground-glass nodule (GGN) has been discovered more and more. Because there are still many uncertainties in the diagnosis and treatment of multifocal GGN in lung, this paper reviews the clinical concerns such as the follow-up interval and time, the relationship between main focus and other focuses, diagnosis, treatment and follow-up of residual nodules.

## 1 总论

### 1.1 肺磨玻璃结节

1996年Fleischner学会提出肺磨玻璃结节(ground-glass nodule, GGN)的概念^[1]^。根据GGN内部是否含有实性成分, 分为纯磨玻璃结节(pure GGN, pGGN)和部分实性结节(part-solid nodule, PSN), 而pGGN及PSN又称为亚实性结节(sub-solid nodule, SSN)^[2-4]^。与实性结节相比, GGN与肺腺癌的关系较为密切, 后者包括原位腺癌(adenocarcinoma *in situ*, AIS)、微浸润腺癌(minimally invasive adenocarcinoma, MIA)和浸润性腺癌^[5]^。

有研究^[6]^发现, GGN大小与肺癌存在相关性, < 6 mm的恶性概率为1%(1/136), 6 mm-10 mm的恶性概率为20%(14/70), 11 mm-20 mm的恶性概率为45%(10/22), > 20 mm的恶性概率为80%(4/5);GGN密度也与肺癌存在相关性, 实性结节的恶性概率仅为7%(14/89), PSN的恶性概率为63%(10/16), pGGN的恶性概率为18%(5/28)。≤10 mm的pGGN大约有25%的概率是AIS和小于5%的概率是浸润性腺癌; > 10 mm的pGGN大约有40%的概率是AIS和20%的概率是浸润性腺癌; ≤10 mm的PSN大约有50%的概率是AIS和25%的概率是浸润性腺癌; > 10 mm的PSN大约有50%的概率是浸润性腺癌。另外, GGN在随访期间大约有20%-30%的概率进展, 多是AIS, 很少有浸润性腺癌。大小有缩小不意味着不需要关注, 除非明显缩小或多次随访^[7]^。

意大利胸外科学会(Italian Society of Thoracic Surgery, SICT)发表了160位成员关于GGN的调查结果^[8]^：高分辨率计算机横断层扫描(high resolution computed tomography, HRCT)检查用于GGN的诊断, 但是40%的受访者也支持常规使用正电子发射计算机横断层扫描(positron emission tomography-CT, PET-CT), 即使是pGGN也是如此。约50%的受访者支持经皮肺穿刺活检, 尤其是 > 1 cm且持续存在或PSN。在初次随访后, 对持续性或不断增长的PSN应行手术切除, 但对持续存在的pGGN是否进行手术仍存在分歧。对实性成分 < 50%的c-I GGN, 亚肺叶切除术优于肺叶切除术, 楔形切除还是肺段切除以及淋巴结清扫仍存在分歧。对实性成分 > 50%的c-I GGN, 亚肺叶切除术劣于肺叶切除术, 同时进行淋巴结清扫。尽管96.2%的受访者认为电视辅助胸腔镜手术(video-assisted thoracoscopic surgery, VATS)可用于治疗pGGN, 但68%的受访者认为VATS仅适用于部分可术中定位的病例。尽管68%的受访者建议对 < 5 mm的pGGN进行影像学随访, 但对CT扫描随访时间和间隔仍存在分歧。

### 1.2 肺多发磨玻璃结节

近年来, 肺多发GGN的诊断越来越频繁, 大约20%-30%切除的GGN病变伴有其他多发较小的肺内GGN病变^[9]^。Hattori等^[10]^研究提示, 肺多发GGN手术切除的病灶多数为腺癌或者癌前病变(占98.9%), 生物学行为属于惰性。但是不应低估pGGN, 因为有高达40%的病例与浸润性腺癌相对应^[9, 11]^。

对国际肺癌研究学会(International Association for the Study of Lung Cancer, IASLC)221位跨学科成员进行了关于肺多发GGN处理的问卷调查^[12]^：63%建议术前获得多个病灶病理, 66%建议做基因检测, 以评估其组织学和基因一致性。63%推荐手术切除(其他18%不建议手术, 19%不确定), 81%的外科医生倾向手术切除, 明显高于肿瘤内科医生的54%、肺科医生的66%和放射肿瘤医生的45%(*P*=0.003, 9);术式以肺叶切除(针对主要病灶)和各种联合肺段切除(针对次要病灶)为主。

虽然已经发布了一些关于肺多发GGN的指南, 但多是基于CT影像的随访标准, 对临床工作几乎没有指导意义, 因为临床更关注GGN的随访间隔与时限、主病灶和次要病灶的关系、穿刺活检、基因检测、治疗和残留结节随访等。

## 2 诊断

### 2.1 随访间隔与时限

研究^[13, 14]^发现30%-90%的pGGN后在随访3个月后消失, 且可以新发50%的pGGN, 这些消失的pGGN考虑是炎症。在新型冠状病毒肺炎(corona virus disease 2019, COVID-19)疫情严重的今天, 更应该加以重视鉴别诊断。所以临床上一般认为随访3个月大小和形状仍没有变化的GGN不除外肺癌的可能。Fleischner学会指南建议对所有多发GGN的病例在3个月-6个月内复查CT, 至少1个GGN > 6 mm, 考虑为多原发肺腺癌^[3]^。

日本一项对78例肺多发GGN患者进行随访的研究^[15]^, 中位随访时间为45.5个月, 随访期内37%增大, 其中大多数在36个月内出现, 因此建议对肺多发GGN患者的最佳观察时限为36个月。

### 2.2 影像学检查

① PET-CT：pGGN病变标准化摄取值(standardized uptake value, SUV)较低, PET-CT检查价值有限, 一般不推荐。PET-CT检查主要用于实性或部分实性结节(实性成分 > 10 mm)^[16-18]^; ②胸部CT增强扫描：pGGN病变原则上不需要做CT增强扫描; 但部分实性结节、病灶与肺血管关系密切或者怀疑淋巴结转移者可行胸部CT增强扫描。通过人工智能(artificial intelligence, AI)辅助诊断系统, 从CT等医学影像图像分析肿瘤生物学特征和影像学特征之间的定量关系, 从而构建肿瘤的诊断、疗效评价及预测等模型; ③分期检查：一般不必做骨扫描、头颅MRI检查和腹部超声等分期检查^[19]^。

### 2.3 病理检查

① 经皮肺穿刺(transthoracic needle aspiration, TTNA)活检：Fleischner学会推荐实性成分≥5 mm做TTNA或手术。活检的原因是无法手术切除或决定手术术式。但是易出血、易气腔内播散、易发生空气栓塞。SSN活检的敏感性为64.6%-96.8%, 与实性成分相关^[20-24]^; ②经气管镜肺穿刺活检：可以经过电磁导航支气管镜(electromagnetic navigation bronchoscopy, ENB)、支气管超声导向鞘(endobronchial ultrasonography with a guide sheath, EBUS-GS)、虚拟支气管镜导航(virtual bronchoscopy navigation, VBN)及衍生出的经肺实质结节隧道(bronchoscopic transparenchymal nodule access, BTNA)穿刺活检。敏感性70%左右, 并发症发生率低。

### 2.4 基因检测

基因检测的目的不是为了治疗, 而是为了诊断。有研究^[25]^显示, 多发GGN在基因和肿瘤发生上具有巨大的异质性, 也就是说均为独立发展的病灶, 病灶之间的基因变异差异很大, 可以肯定是多原发而不是转移。如果仅计算表皮生长因子受体(epidermal growth factor receptor, EGFR), 则在整个人群中, 驱动基因突变的不一致率是80%左右^[26, 27]^。驱动基因突变进化树显示：不同区域肿瘤呈现分支进化, 解剖学位置相距较远的肿瘤间突变特征差异更明显。在同一信号通路上的驱动事件呈异质性, 但在生物学功能上是趋同的。肿瘤形成过程积累突变不同, 进化压力对基因的多样性兼具扩展和约束的机制^[25]^。

## 3 治疗

根据GGN的解剖位置、大小和数量, 可以考虑亚肺叶切除和肺叶切除, 双侧病变可以考虑同期或分期手术。肿瘤热消融是肺多发GGN的治疗方法之一。

### 3.1 适应证

① 高危因素：中老年人(55岁-74岁)、既往恶性肿瘤病史、家族史、长期吸烟史(> 30年, 或戒烟年限 < 15年)、或特殊职业接触史(石棉)等情况; ②影像学上恶性征象：毛刺征、分叶征、胸膜凹陷、部分实性; 动态观察发现GGN增大、实性成分增加; 贴近脏层胸膜的周围型GGN可局部切除。主病灶最大径和肿瘤实性成分比值(consolidation tumor ratio, CTR)是医师判断结节良恶性和手术时机的参考依据; ③患者极度焦虑, 无法缓解。

### 3.2 术前辅助定位

① 术前定位技术：CT引导下经皮肺穿刺注射医用胶、亚甲蓝、吲哚菁绿(ICG)等液体材料, 或放置微弹簧圈、Hook-wire等辅助定位; 经电磁导航支气管镜或虚拟支气管镜导航注入染料等定位^[28]^; ②术中定位技术：术中B超定位、术中立体解剖定位。

### 3.3 胸腔镜手术

(1) 切口选择：GGN首选治疗方式是胸腔镜手术, 包括单孔胸腔镜、二孔胸腔镜、三孔胸腔镜、剑突下胸腔镜等; (2)手术原则：①主病灶优先, 兼顾次病灶; ②同一肺叶双原发或多原发结节：同期手术多采用肺叶切除; ③同侧不同肺叶单发病灶：若患者肺功能允许, 可采取同期手术, 一般较大病灶所在的肺叶行肺叶切除术, 小病灶采取肺楔形切除; 若两病灶较小, 可采用不同肺叶的亚肺叶切除; ④当病灶分别位于两侧肺叶时, 选择分期切除的手术原则是：a.先切除中心型、进展较快、病灶较大或伴有纵隔、肺门淋巴结转移的主病灶, 后切除周围型、进展较慢、病灶较小或无淋巴结转移的其他病灶; b.先切除对预后影响较大的病灶：如病灶较大、密度较高、实性成分较大、恶性征象明显、分期较晚的病灶; c.两次手术间隔时间太短不利于患者初次手术后的恢复, 增加二次手术的风险; 而间隔时间太长又会增加未切除侧病灶进展和转移的风险, 一般两次手术的时间间隔应在4周-6周; ⑤选择同期切除遵循的手术原则是：a.安全：先进行切除范围小的一侧, 确保对侧手术安全; b.不安全：先切除主病灶, 二期对侧手术; ⑥关于淋巴结清扫：CTR是淋巴结转移的重要预测因素, 回顾性研究^[29, 30]^发现, CTR < 0.5的肿瘤没有肺门或纵隔淋巴结转移, CTR≥0.5的肿瘤中有10%发生了转移。Ye等^[31]^回顾性研究发现, 55例pGGN没有淋巴结转移, 292例PSN有6例发生了N1淋巴结转移, 3例发生了N2淋巴结转移。AAH、AIS、MIA、贴壁为主型腺癌和浸润性黏液腺癌没有淋巴结转移。无论采取何种手术方式, 系统淋巴结采样都是必要的, 它对延长肿瘤局部控制时间、提高治愈率及完善诊断分期均具有重要意义^[32, 33]^。

多灶性GGN/贴壁生长肿瘤切除术后5年生存率在90%以上, 即使是亚肺叶切除也不影响预后^[17]^。

### 3.4 肿瘤热消融

Kodama等^[34]^回顾性评价了射频消融治疗33例患者的42个GGN优势(≥50.0%)肺腺癌的临床结果, 平均随访42个月, 局部进展率14.3%(6/42), 6例中的4例再次消融, 除1例脑出血死亡以外, 均存活, 1年总生存率和肿瘤特异性生存率分别为100.0%和100.0%, 3年分别为96.4%(95%CI: 77.5%-99.5%)和100.0%, 5年分别为96.4%(95%CI: 77.5%-99.5%)和100.0%。Iguchi等^[35]^回顾性评价了射频消融治疗16例患者的17个表现为GGN为主(≥50.0%)肺癌的临床结果, 中位肿瘤随访61.5个月, 首次和二次技术效率1年分别为100.0%和100.0%, 2年分别为93.3%和100.0%, 3年分别为78.3%和92.3%;中位患者随访65.6个月, 1例患者11.7个月因其他癌症复发而死亡, 其余16例均存活, 1年总生存率和肿瘤特异性生存率分别为93.3%和100.0%, 5年分别为93.3%和100.0%。Yang等^[36]^回顾性分析了微波消融治疗肺部周围型磨玻璃密度影(ground-glass opacity, GGO)(腺癌)的初步结果, 51例肺部GGO患者经微波消融治疗后3年的无局部复发生存率、肿瘤特异性生存率和总生存率分别为98.0%、100.0%和96.0%。

### 3.5 术后处理

多数GGN术后病理提示早期肺腺癌的不需要化疗和放疗。只有极少数部分实性结节, 如果病灶较大, 或者合并淋巴结转移才需要化疗。目前研究还没有证实分子靶向药物治疗对GGN患者有好处, 除非因为肺功能低下而无法切除时, 才建议患者做基因检测, 以防将来复发时考虑靶向治疗。同期多数原发GGN如因肺功能因素无法行根治性切除时, 可考虑化疗。

### 3.6 残留病灶

肺多发GGN的每个病灶都是独立的, 有观点认为主病灶影响预后, 也有观点认为不仅要看主病灶, 还要看其他病灶的大小和实性成分, 尤其是实性成分的改变。日本的一项研究^[16]^发现, 如果主病灶实性成分为主或 > 25 mm, 则其预后(5年OS)较GGN为主且大小≤25 mm者差。国际肺癌研究协会(International Association for the Study of Lung Cancer, IASLC)分期委员会对CT上表现肺多发GGN病变的腺癌亚型进行了综述^[17]^, 尽管该综述的重点是组织学证实为腺癌的患者, 而不是筛查检查到的肺多发GGN, 但得到了类似的结论, 即多灶性GGN/贴壁生长肿瘤的预后很大程度上取决于主病灶在肿瘤原发灶-淋巴结-转移(tumor-node-metastasis, TNM)分期系统中最高T分期。但是, 日本针对246例多发GGN患者的研究发现, 主病灶和其他病灶都会影响生存率^[18]^。Shimada等^[37]^对67例肺多发GGN手术切除(39例完全切除)的回顾性研究发现：未切除GGN仅8%变化, 23%患者出现新发病灶; 多因素分析发现主病灶大小和实性成分与预后相关, 次要或残留病灶及是否生长、新发不影响预后。其他研究^[38-40]^也证实, 当切除主病灶后, 无论其他病灶继续生长, 还是出现新的病灶, 或剩余病灶未予处理, 都不会影响患者的OS。

总之, 肺GGN中约30%为多发性的, 且至少需要随访3个月, 大小没有变化或增大才需要干预, 不接受干预者至少随访3年, 对部分缩小者也不能掉以轻心。基因检测发现各个病灶间存在明显的基因异质性, 证实肺多发GGN是多原发肿瘤而不是转移。肺多发GGN的手术切除效果非常好, 但是受到肿瘤热消融技术的挑战(图 1)。

**1:**
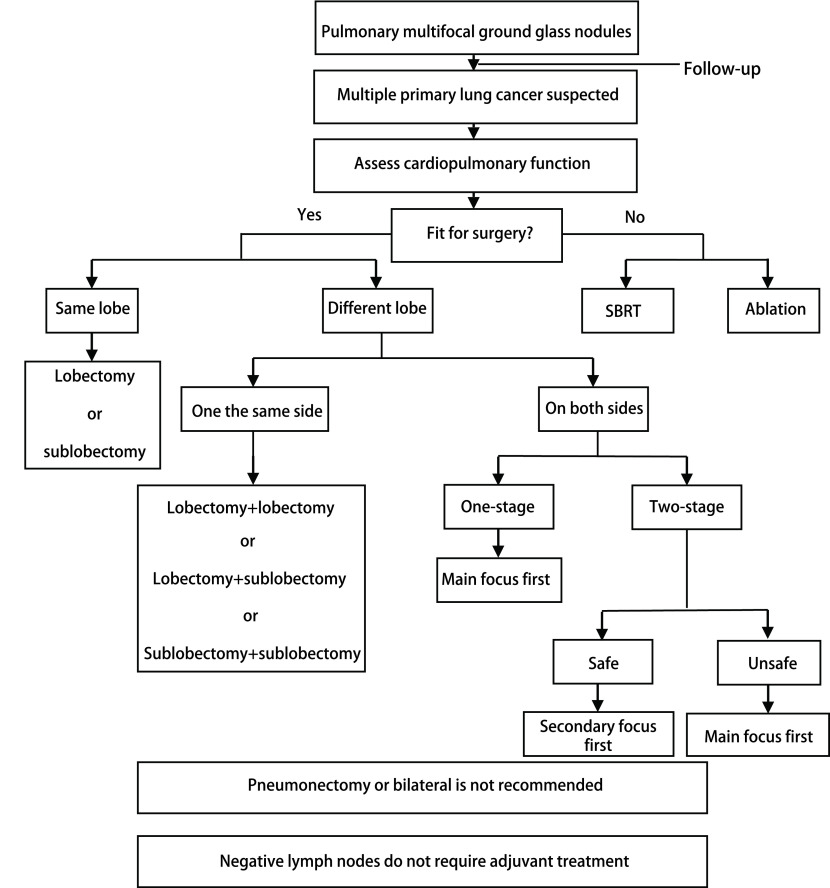
肺多发磨玻璃结节诊治流程 Flow chart of diagnosis and treatment of pulmonary multifocal ground glass nodules. SRBT: stereotactic radiotherapy.
